# Calcifications in triple-negative breast cancer: Molecular features and treatment strategies

**DOI:** 10.1038/s41523-023-00531-4

**Published:** 2023-04-15

**Authors:** Cai-Jin Lin, Wen-Xuan Xiao, Tong Fu, Xi Jin, Zhi-Ming Shao, Gen-Hong Di

**Affiliations:** Department of Breast Surgery, Fudan University Shanghai Cancer Center; Key Laboratory of Breast Cancer in Shanghai, Department of Oncology, Shanghai Medical College, Fudan University, Shanghai, 200032 China

**Keywords:** Breast cancer, Predictive markers, Cancer genomics, Cancer microenvironment, Cancer metabolism

## Abstract

Despite the high prevalence of mammographic calcifications, our understanding remains limited regarding the clinical and molecular features of calcifications within triple-negative breast cancer (TNBC). To investigate the clinical relevance and biological basis of TNBC with calcifications of high suspicion for malignancy, we established a study cohort (*N* = 312) by integrating mammographic records with clinical data and genomic, transcriptomic, and metabolomic profiling. Despite similar clinicopathological features, patients with highly suspicious calcifications exhibited a worse overall survival than those without. In addition, TNBC with highly suspicious calcifications was characterized by a higher frequency of *PIK3CA* mutation, lower infiltration of immune cells, and increased abnormality of lipid metabolism. Overall, our study systematically revealed clinical and molecular heterogeneity between TNBC with or without calcifications of high suspicion for malignancy. These data might help to understand the clinical relevance and biological basis of mammographic calcifications.

## Introduction

Triple-negative breast cancer (TNBC), accounting for approximately 15% of breast malignancies, is both clinically and biologically heterogeneous and characterized by aggressive behavior and a paucity of effective treatments, leading to TNBC being the subtype with the least favorable prognosis^1,2^. Impressive progress in cataloging the molecular basis of TNBC has been achieved from the perspectives of transcriptomics, immunogenomics, and metabolomics^3–8^. The new appreciation of the molecular biology of TNBC revolutionizes the therapeutic landscape and provides new therapeutic options, such as immune checkpoint blockade (ICB), PARP inhibitors, and PI3K inhibitors^2,9^. Although the new therapeutic scenario has full potential to improve outcomes, treatment response varies, and thus, a better understanding of the intrinsic and extrinsic features of TNBC is required to extend the clinical benefit.

Mammography has been established as one of the periodical screening modalities since it demonstrates higher sensitivity toward breast cancers primarily manifesting as calcifications^10,11^. Several studies suggest an association of malignant calcifications with clinicopathological features and patient prognosis^12–17^. It was also reported that mammographic calcifications were more prevalent in non-TNBC tumors^18–21^. However, approximately 10–30 percent of TNBCs present calcifications^20–24^. In addition, the presence of calcifications was associated with increased mortality rates and decreased chemotherapy responsiveness for TNBC patients^25–28^, indicating the necessity to explore the biological basis of TNBC with calcifications. Despite a growing focus, elaborating the heterogeneity regarding the molecular biology, clinical outcomes, and potential therapeutic response between TNBC with or without malignant calcifications has lagged behind. This could be partially attributed to the paucity of studies linking mammographic features to multiomics profiling data, contributing to the malignant calcifications in TNBC being a prevalent yet poorly understood clinical issue.

These challenges necessitated the broader interpretation of the molecular basis for TNBC with malignant calcifications. To address this issue, we established the largest mammographic multiomics cohort by integrating mammographic images with genomic, transcriptomic, and metabolomic profiling as well as detailed health records to shed new light on the clinical and biological heterogeneity between TNBC with or without calcifications of high suspicion for malignancy, and thus, help inform treatment decisions.

## Results

### Study cohort and clinical data

**Fig. 1:**
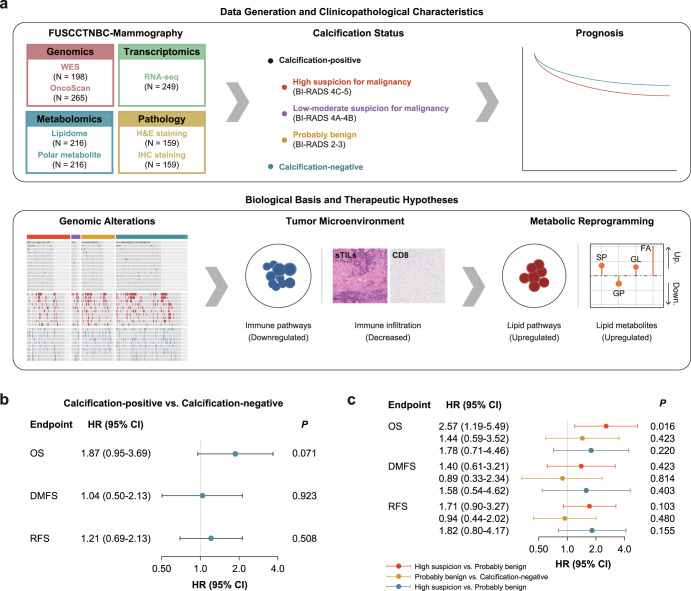
Schematic overview of the study design. **a** Omics platforms and analysis workflow of the study. **b** Prognostic value of mammographic calcifications. **c** Prognostic value of calcification of high suspicion for malignancy. Error bars represent confidence intervals of the corresponding hazard ratios for different clinical outcomes. Cox regression models were used to estimate the hazard ratios and corresponding confidence intervals.

The FUSCC-Mammography cohort included a total of 312 patients diagnosed with TNBC with a median follow-up of 86.4 months (interquartile range: 68.7–101.6 months; Fig. 1a). Clinical records (*N* = 312) and mammographic images (*N* = 312) were collected in detail, coupled with hematoxylin and eosin (H&E) and immunohistochemical (IHC) staining slides (*N* = 159), whole exome sequencing (WES; *N* = 198), OncoScan (*N* = 265), transcriptomics (*N* = 249), lipidomics and polar metabolomics (*N* = 216) data.

We reviewed their mammographic images and determined the status of mammographic calcifications based on the Breast Imaging Reporting and Data System (BI-RADS)^29,30^. Based on the BI-RADS categories, we divided patients with calcifications (calcification-positive) into three groups representing different suspicions for malignancy: patients with mammographic calcifications assessed as BI-RADS 4C-5 were categorized as high suspicion for malignancy; patients with calcifications assessed as BI-RADS 4A-4B were categorized as low-moderate suspicion; patients with calcifications assessed as BI-RADS 2-3 were categorized as probably benign. Patients without calcifications were categorized as calcification-negative. Due to the limited sample size of patients with low-moderately suspicious calcifications, we mainly focused on the difference between highly suspicious and probably benign calcifications or between highly suspicious calcifications and calcification-negative tumors.

We first compared clinicopathological characteristics and observed similar distribution, except a higher proportion of architectural distortion within highly suspicious calcifications group (Supplementary Table 1). Despite clinicopathological homogeneity, we observed that the association of calcifications with overall survival (OS) trended toward significance (Fig. 1b). Further analysis revealed a significant association between highly suspicious calcifications and OS but not distant metastasis-free survival (DMFS) or relapse-free survival (RFS) (Fig. 1c). These data suggest a potential biological basis underlying the prognostic difference and necessitate further investigation.

Overall, we established a mammographic multiomics cohort and found an association of clinical outcomes with highly suspicious calcifications, suggesting further investigation of the underlying biological basis.

### Molecular landscape of the FUSCCTNBC-Mammography cohort

**Fig. 2:**
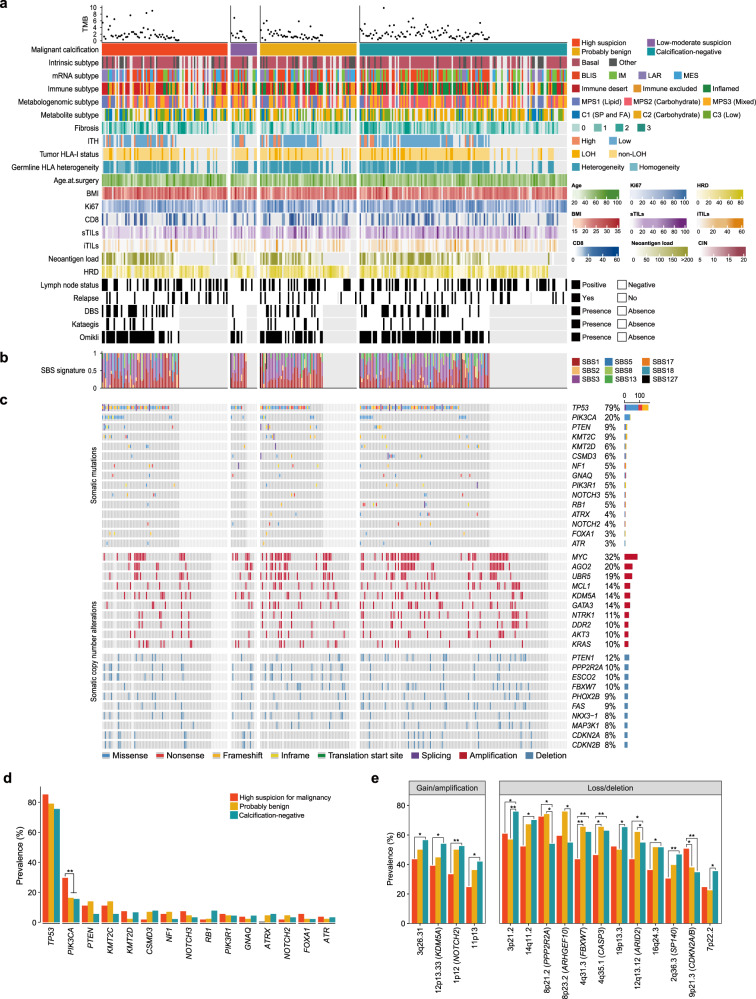
Molecular landscape of the FUSCCTNBC-mammography cohort. **a** Three hundred and twelve TNBC samples annotated with clinical and molecular features. Samples are ordered by calcification status. **b** Somatic mutations of the top mutated genes. Genes are ordered by the total mutation frequencies. **c** Copy number alteration of cancer-related genes. Only amplifications (GISTIC + 2) and deep deletions (GISTIC -2) are presented. **d** Gene-level mutation frequencies across different calcification groups. “**” denotes a *P*-value of < 0.01. **e** Region-level SCNA frequency across different calcification groups. “**” denotes a *P*-value of < 0.01 and “*” denotes a *P*-values of < 0.05. Logistics regression models using the binomial family were performed to obtain *P* values for the comparison analyses of mutations and SCNAs between different calcification groups.

Here, we presented a well-annotated landscape of the FUSCCTNBC-Mammography cohort (Fig. 2a–c). Across the FUSCCTNBC-Mammography cohort with WES data, 14,974 protein-altering and splice site variants were identified, comprising 13,925 single nucleotide variants (SNV) and 1049 insertions or deletions (INDEL), and a median of 53 SNVs and 3 INDELs were found per tumor. Genes most frequently mutated or amplified/deleted are presented in Fig. 2b, c.

We first investigated the difference in mutational profiling and found that *PIK3CA* was mutated more frequently in patients with calcifications of high suspicion for malignancy (29.6% in high suspicion vs. 16.3% in probably benign vs. 15.6% in calcification-negative; Fig. 2d). We then deciphered underlying mutational processes across different calcification groups. We observed subtle variations in the mutational spectra of somatic substitutions (Supplementary Fig. 1a–d). Tumors with highly suspicious calcifications were characterized by decreased C > G (Supplementary Fig. 1e). We then deconvoluted breast cancer-specific mutational signatures and found that tumors with highly suspicious calcifications exhibited increased activity of the single-base substitution (SBS) signature SBS17 (Supplementary Fig. 1f). For SBS17, the etiology remains unknown but may be associated with reactive oxygen species damage in some cases^31^ or signatures of mismatch repair defects^32^. This might suggest distinct mutagenesis across different calcification groups but cautious interpretation and further validation are needed. Overall, our study established the connection between highly suspicious calcifications and *PIK3CA* mutation, which should be considered when administering PI3K inhibitors to potential candidates.

### The association of highly suspicious calcifications with recurrent copy number alterations

Given that somatic copy number alterations (SCNA) are prevalent in TNBC, we concentrated on the association between SCNAs and calcifications. We ran GISTIC2 to estimate the recurrently amplified and deleted regions of interest (ROIs) and observed slightly different global SCNA patterns between different calcification groups (Supplementary Figure 2). However, differences existed in specific recurrent ROIs, especially in copy number losses and deletions (Fig. 2e). For example, we found a lower gained/amplified frequency of 1p12 (*NOTCH2*) in highly suspicious calcifications than in probably benign calcifications and calcification-negative tumors (33.3% in highly suspicious vs. 50.0% in probably benign vs. 52.4% in calcification-negative). Similarly, we also found a lower frequency of 4q31.3 (*FBXW7*) losses/deletions in highly suspicious calcifications (43.5% in highly suspicious vs. 65.5% in probably benign vs. 62.1% in calcification-negative). Both the oncogene of *NOTCH2* amplification and the tumor suppressor of *FBXW7* deletion are involved in the NOTCH oncogenic pathway and indicate a possible involvement of NOTCH signaling in TNBC with highly suspicious calcifications^33^. In addition, we observed that TNBC with highly suspicious calcifications exhibited a higher deletion of 9p21.3 (*CDKN2A/B*; 50.7% in highly suspicious vs. 37.9% in probably benign vs. 34.7% in calcification-negative), revealing potential activation of the cell cycle pathway^33^. The association of highly suspicious calcifications and recurrent CNAs with involvement of specific pathways was speculated and should be interpreted cautiously since no experimental validation was performed.

Afterward, we examined SCNA signatures and found significantly higher activity of signature CN6 in tumors with highly suspicious calcifications when compared with probably benign calcifications and calcification-negative tumors (Supplementary Fig. 1f). CN6 is associated with chromothripsis before genome doubling and patients with the CN6 signature exhibited poorer prognosis^34^, further supporting unfavorable outcomes in TNBC with highly suspicious calcifications. Taken together, tumors with different statuses of calcifications presented distinct SCNA patterns.

### The role of calcifications in microenvironment phenotypes

**Fig. 3:**
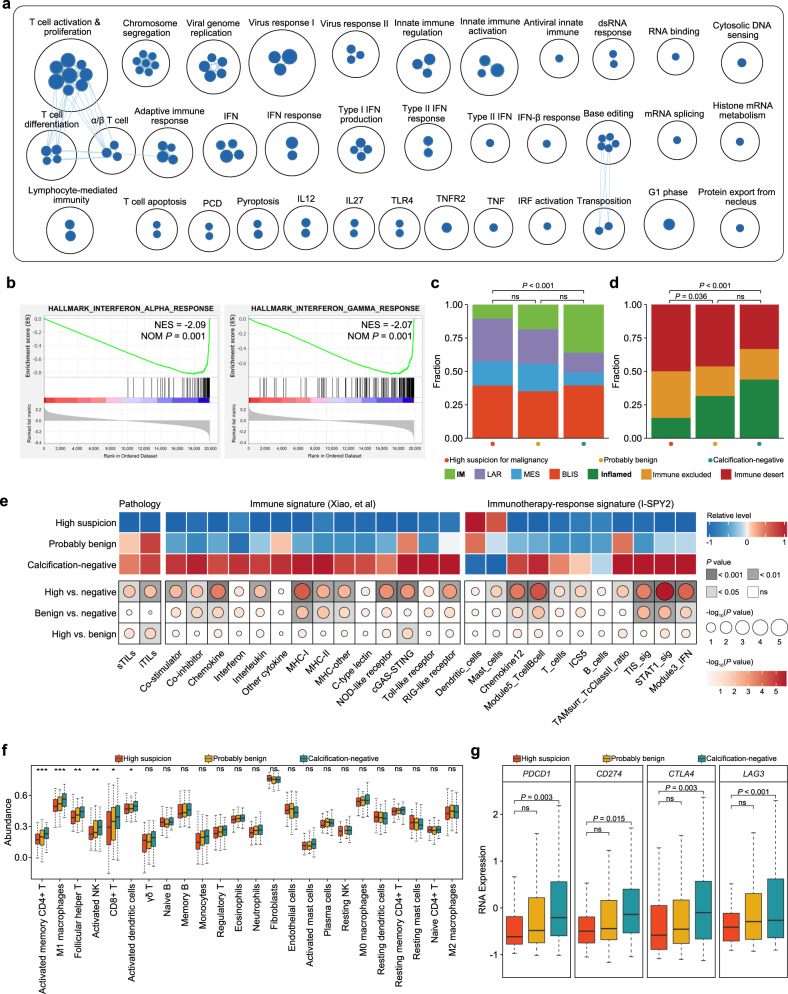
Tumor microenvironment phenotypes across different calcification groups. **a** An overview of gene circuits downregulated in tumors with calcifications of high suspicion for malignancy by mRNA abundance. Nodes represent pathways and edges represent shared genes between pathways. **b** GSEA showing the downregulated interferon-related pathways within tumors with calcifications of high suspicion for malignancy. **c**, **d** The association of mammographic calcifications with the TNBC mRNA subtype (**c**) and immune subtype (**d**). “ns” denotes a *P*-value of > 0.05. **e** Comparison of the sTILs, iTILs, literature-defined immune signatures, and immunotherapy-response signatures across different calcification groups. **f** Inferred immune cell infiltrates across tumors with different statuses of calcifications. The center lines represent median values; the bounds of the boxplot represent the interquartile ranges; the whiskers show the range of the data. “***” denotes a *P*-values of < 0.001; “**” denotes a *P*-value of < 0.01; “*” denotes a *P*-values of < 0.05; “ns” denotes a *P*-value of > 0.05. **g** Comparison of the expression of *PDCD1*, *CD274*, *CTLA4*, and *LAG3* across different calcification groups. “ns” denotes a *P*-value of > 0.05. The center lines represent median values; the bounds of the boxplot represent the interquartile ranges; the whiskers show the range of the data. All *P* values were obtained based on logistics regression models with the binomial family used for categorical data (**c**, **d**) and the gaussian family used for continuous data (**e**–**g**).

We further investigated the association between calcifications and microenvironment compositions. Pathway enrichment analysis demonstrated downregulation of multiple immune-related pathways in tumors with calcifications of high suspicion for malignancy (Fig. 3a). For example, interferon (IFN) signaling was significantly downregulated (Fig. 3b). Consistently, TNBC with highly suspicious calcifications harbored fewer immunomodulatory or inflamed subtypes but more immune-excluded or immune desert subtypes (Fig. 3c, d), which was also validated by decreased levels of stromal tumor-infiltrating lymphocytes (sTILs) and intra-tumoral TILs (iTILs) (Fig. 3e). We also found that tumors with highly suspicious calcifications presented globally lower scores of several literature-defined immune signatures (Fig. 3e), further supporting an immunosuppressive microenvironment in TNBC with calcifications of high suspicion for malignancy.

We then investigated the extrinsic mechanisms of immune escape in tumors with different calcification statuses^35^. Pathway enrichment analysis revealed inactivation of innate immune immunity (Fig. 3a). Similarly, several sensors of nucleic acids that initiate innate immunity, such as cGAS-STING proteins and RIG-like and NOD-like receptors, were also downregulated (Fig. 3e). Lower expression of *IFNG* (Supplementary Figure 3a) and downstream IFN-responsive genes, including *CXCL9/10/11* (Supplementary Figure 3b–d), also supported these findings. Lower expression of these molecules might disrupt the chemotaxis of innate and adaptive immune cells within tumors with highly suspicious calcifications. The inferred immune cell fraction also supported a lower immune infiltration within tumors with highly suspicious calcifications, including CD4 + , CD8 + T cells, and NK cells (Fig. 3f).

Likewise, we also explored the potential intrinsic immune evasion mechanisms, which refers to tumor cells facilitating immune escape themselves. Tumor immunogenicity and immune checkpoint molecule expression comprise two main mechanisms^35^. To assess tumor immunogenicity, we compared germline HLA homogeneity (Supplementary Figure 3e), tumor HLA-I status (Supplementary Figure 3f), intra-tumoral heterogeneity level (Supplementary Figure 3g), tumor mutation burden (Supplementary Figure 3h), chromosome instability score (Supplementary Figure 3i), and neoantigen load (Supplementary Figure 3j), but did not observe any evident disparities, indicating no significant difference in neoantigen source. However, the expression levels of the MHC-I and MHC-II families were significantly lower in tumors with highly suspicious calcifications (Fig. 3e), suggesting the potential inability to present antigens for tumors with highly suspicious calcifications.

To investigate the potential response to immune checkpoint blockade (ICB) for tumors with highly suspicious calcifications, we compared the expression levels of several immune checkpoints and immune-related signatures derived from the I-SPY2 trial^36^. We observed lower expression of *PDCD1*, *CD274, CTLA4, and LAG3* within the highly suspicious calcification group (Fig. 3g). Consistently, tumors with highly suspicious calcifications also scored lower on multiple immunotherapy-response signatures, such as TIS_sig and STAT1_sig (Fig. 3e). For the immunotherapy-resistance signature in the I-SPY2 trial, namely Mast_cells, tumors with highly suspicious calcifications scored higher than calcification-negative tumors. These data might suggest worse efficacy of immunotherapy for patients with calcifications of high suspicion for malignancy.

Altogether, we identified the potential extrinsic and intrinsic mechanisms of immune evasion for TNBC with calcifications of high suspicion for malignancy and estimated a potentially worse response to ICB treatment.

### Metabolomic analyses suggest an enrichment of lipid metabolism in tumors with calcifications

**Fig. 4:**
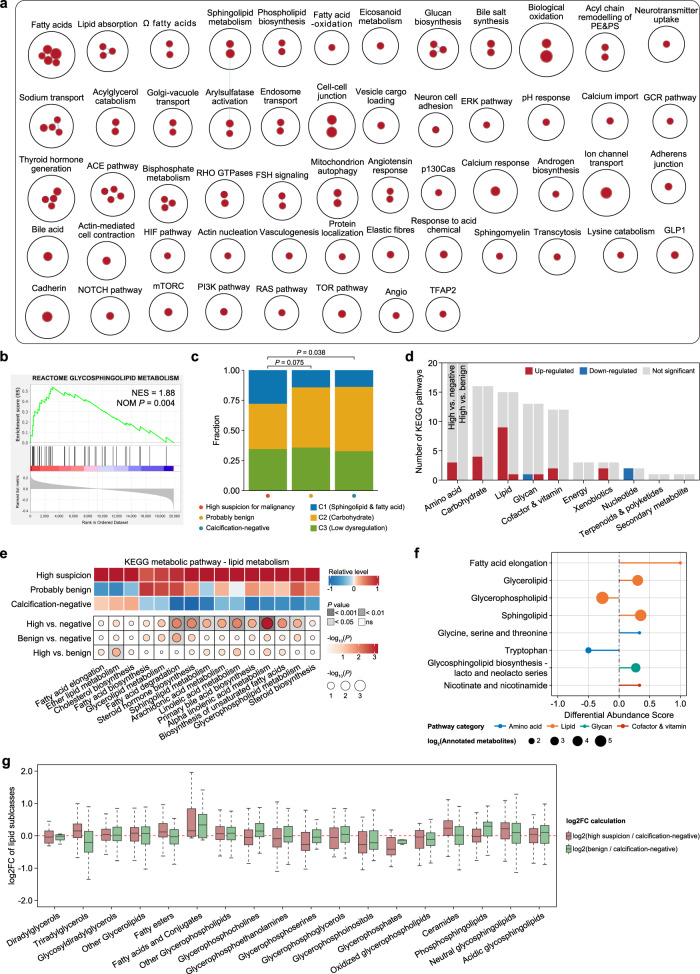
Association of mammographic calcifications with metabolic dysregulation. **a** An overview of gene circuits upregulated in tumors with calcifications of high suspicion for malignancy by mRNA abundance. Nodes represent pathways, and edges represent shared genes between pathways. **b** GSEA showing the upregulated pathways involving lipid metabolism within tumors with calcifications of high suspicion for malignancy. **c** Association of mammographic calcifications with the TNBC metabolite subtype. *P* values were obtained based on logistics regression models using the binomial family. **d** The number of metabolic pathways that were significantly upregulated or downregulated across different calcification groups. **e** Comparison of the signatures involving lipid metabolism across different calcification groups. *P*-values were obtained based on logistics regression models using the gaussian family. **f** A pathway-based analysis of metabolomic changes across different calcification groups using Mann–Whitney U tests. The DA score represents the average and gross changes for all metabolites within a single pathway. A score of 1 indicates that all profiled metabolites of the pathway are upregulated in the calcification-positive group compared to the calcification-negative group, and a score of −1 indicates that all profiled metabolites of a pathway are downregulated. Only pathways with no less than three profiled metabolites were used for DA score calculation. **g** Log_2_ fold changes in the abundances of lipids subclasses in tumors with different calcification statuses. The center lines represent median values; the bounds of the boxplot represent the interquartile ranges; the whiskers show the range of the data.

Since metabolic reprogramming is an established cancer hallmark, we then characterized the metabolic heterogeneity between tumors with and without calcifications of high suspicion for malignancy. Enrichment analysis demonstrated upregulation of pathways related to lipid metabolism in tumors with highly suspicious calcifications (Fig. 4a, b). Consistently, for the metabolite-based subtype, TNBC with highly suspicious calcifications harbored a more lipid-dysregulated subtype (Fig. 4c). When focusing on metabolic pathways, we also observed that most dysregulated metabolic processes were associated with lipid metabolism (Fig. 4d), further supporting the association of lipid metabolism with highly suspicious calcifications. We then presented the activity of 15 KEGG lipid-related pathways and observed a globally higher score for the highly suspicious group (Fig. 4e). Furthermore, KEGG metabolic pathway-based differential abundance (DA) analysis was conducted between tumors with highly suspicious calcifications and the rest of tumors to determine the dysregulation of metabolic pathways in terms of metabolites. Likewise, tumors with highly suspicious calcifications obtained a higher DA score of pathways involved in fatty acid, sphingolipid, and glycerolipid metabolism (Fig. 4f), indicating an upregulated metabolism of the corresponding metabolites. When focusing on lipid subclasses, we observed an increased metabolism of triradylglycerols, fatty esters, fatty acids, ceramides, and neutral glycosphingolipids (Fig. 4g). Collectively, tumors with calcifications of high suspicion for malignancy displayed distinct metabolic patterns and were characterized by lipid dysregulation, indicating the potential benefits from lipid regulators.

## Discussion

**Fig. 5:**
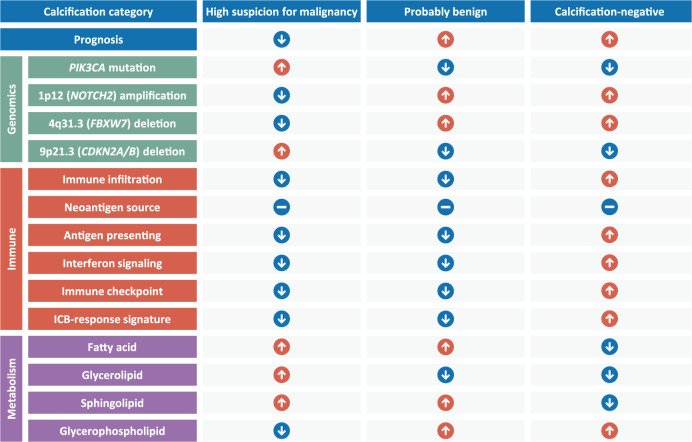
Summary of the molecular characteristics across TNBC with different calcification statuses. Red (blue) circles with white up (down) arrows denote increased (decreased) levels or potentially better (worse) response, while the blue circles with horizontal bars indicate no significant difference.

To shed light on the clinical and molecular heterogeneity between TNBC with or without calcifications of high suspicion for malignancy, we established a large-scale mammographic multiomics cohort thus far and summarized the corresponding molecular characteristics (Fig. 5). Despite similar clinicopathological features, we found that patients with calcifications of high suspicion for malignancy exhibited a worse prognosis, further supporting the investigation of the biological basis. In addition, tumors with highly suspicious calcifications were also characterized by a higher likelihood of *PIK3CA* mutation, lower immune infiltration and inability of antigen presentation, and increased lipid metabolism, indicating potential resistance to ICB and benefit from PI3K inhibitors or lipid regulators.

We first concentrated on the clinicopathological features and prognosis. In contrast to previous findings^37,38^, we found a similar distribution of conventional clinicopathological characteristics between TNBC with or without highly suspicious calcifications, except for higher architectural distortion. However, despite the similar clinicopathological features, patients with highly suspicious calcifications presented worse prognosis than those without, suggesting a more aggressive behavior, which is also reported in other publications^12–16,26,39^. All these findings indicate the potential clinical homogeneity between TNBC with and without calcifications of high suspicion for malignancy, thus necessitating a focus on molecular heterogeneity, which is an emphasis in our study to account for the prognostic difference.

In our study, we found that TNBC with highly suspicious calcifications exhibited increased *PIK3CA* mutations, which was significantly higher than the rest of TNBC and the frequency in the general TNBC population reported by previous studies^3^. To the best of our knowledge, this is the first study that associated highly suspicious calcification with increased likelihood of *PIK3CA* mutation. In addition, we also found that TNBC with highly suspicious calcifications constituted a higher fraction of the luminal androgen receptor subtype, which was reported to exhibit the highest *PIK3CA* mutation frequency among the TNBC subtypes^3,40^. All these data suggested that calcifications of high suspicion for malignancy might serve as a predictive biomarker for *PIK3CA* mutation and treatment response to PI3K inhibitors.

The association of mammographic calcifications with microenvironment compositions was further explored and we found that tumors with calcifications of high suspicion for malignancy exhibited an inhibitory immune microenvironment. Very few studies have investigated the association of mammographic calcifications with the tumor microenvironment. Shin et al. revealed that immune and inflammatory responses were downregulated in breast cancers with calcifications^39^. However, this study focused only on the differentially expressed genes and observed an enrichment of immune-related pathways. In our study, we further investigated the association of highly suspicious calcifications with the microenvironment from both extrinsic and intrinsic perspectives and validated the finding through pathologically evaluated TILs infiltration. We observed globally decreased expression levels of immune-related molecules involved in IFN signaling, chemotaxis of immune cells, and antigen presentation as well as lower scores for immunotherapy-response signatures. Collectively, these data might suggest that mammographic calcifications should be considered when determining immunotherapy candidates since tumors with highly suspicious calcifications might exhibit potential resistance to immune checkpoint blockade.

Since the metabolic features of breast cancer have been extensively investigated^5–7^, we also concentrated on the impact of calcifications of high suspicion for malignancy on metabolic profiles. Among the ten KEGG metabolic pathways, we observed a dysregulation of lipid metabolism in tumors with highly suspicious calcifications. Further analysis revealed that tumors with highly suspicious calcifications presented an improved metabolism of fatty acids, glycerolipids, and sphingolipids. While the role of *PIK3CA* mutations in lipid metabolism has also been confirmed in prior work^41–43^, the upregulated lipid metabolism might be potentially attributed to the increased *PIK3CA* mutation in TNBC with highly suspicious calcifications. More specifically, we associated the highly suspicious calcifications with the metabolism of different lipid subclasses, including ceramides, triradylglycerols, and fatty esters. Such an association has never been reported previously and the underlying biological basis requires further investigation.

Our research comprehensively investigates the role of calcifications of high suspicion for malignancy in genome instability, immune evasion, and metabolic reprogramming based on multiomics data. Nevertheless, our work has several limitations. First, due to the limited sample size and non-randomized nature of the study cohort, potential selection bias and other unmeasured confounding bias might exist. Further external validation is required in larger prospective mammographic cohorts. In addition, experiments are needed to validate several observations and hypotheses, for instance, lipid dysregulation and immunoinhibitory microenvironment within tumors with highly suspicious calcifications. Finally, it has been reported that TNBC often lacks mammographic calcifications and other mammographic features, which might contribute to the subtle difference between tumors with and without calcifications in some aspects in our study. We acknowledge the value and have planned further studies in ER-positive and/or HER2-positive breast cancers, which would possibly generate more significant differences and provide clearer insight into the biological basis of mammographic calcifications.

Collectively, we found that TNBC with or without calcifications of high suspicion for malignancy exhibited potential clinical homogeneity but molecular heterogeneity. We identified the association of highly suspicious calcifications with genome instability, immune evasion, and metabolic reprogramming.

## Methods

### Patient samples and study cohorts

We established a cohort, termed FUSCC-Mammography, to include females diagnosed with TNBC, along with preoperative mammography, samples from primary tumors, adjacent tumor tissues, and paired blood samples obtained from Fudan University Shanghai Cancer Center (FUSCC) (Supplementary Table 2). The clinical data, including demographics, postoperative pathology, treatment regimen, and follow-up, were recorded in detail. All women underwent surgery between 2007 and 2014. We then updated the follow-up data on June 30, 2021. All tissue samples included in this study were obtained after the approval of our research by the FUSCC Ethics Committee, and each patient provided written informed consent prior to participation.

Mammographic images as well as H&E- and IHC-stained slides were collected. Detailed information on biospecimen collection, and data generation of WES, OncoScan, transcriptomics, lipidomics, and polar metabolomics were described in previous studies^3,5^.

### Annotation of somatic oncogenic alterations

We applied oncokb-annotator to annotate the oncogenic mutations and SCNAs curated in the OncoKB database^44^. A MAF file containing the mutations annotated by Ensembl Variant Effect Predictor (VEP) and an all-thresholded-by-genes file from GISTIC 2.0 were used as the inputs.

### Generation of mutational matrices

Mutational matrices of SBS96, DBS78, and ID83 based on the somatic mutations and their immediate sequence context were first created using SigProfilerMatrixGenerator with default parameters^45^.

### Mutational signature analysis

We employed SigProfiler^46^, a well-established computational algorithm based on nonnegative matrix factorization (NMF), to extract mutational signatures across tumor samples in the FUSCCTNBC-Mammography cohort with default parameters. Mutational signatures of single base substitutions (SBS), doublet base substitutions (DBS), and small insertions and deletions (ID) were deciphered separately. Specifically, mutational matrices of SBS96, DBS78, and ID83 based on the somatic mutations and their immediate sequence context were first created using SigProfilerMatrixGenerator with default parameters. The mutational matrices were then utilized as the inputs of SigProfilerExtractor for de novo extraction of mutational signatures. NMF was employed with factorizations between *k* = 1 and *k* = 25 and the number of iteration for each factorization of 500. SigProfilerExtractor determined the optimum number of signatures automatically.

We decomposed the mutational matrices of each patient into a known set of reference signatures using SigProfilerSingleSample^46^. The breast cancer-specific reference signatures of SBS and DBS were obtained from Signal Project^32,47^ while ID were downloaded from COSMIC Portal^46^.

### Detection of clustered mutations

Clustered mutations were detected by analyzing the inter-mutational distances (IMD) between SNV-SNV mutations^45^. Specifically, SigProfilerSimulator^48^ was first used to calculate an IMD threshold by comparing the mutational patterns of a given sample between real and simulated data to ensure the clustered events are unlikely to occur by chance. We simulated all somatic mutations in each sample for 100 times and determined the IMD threshold with *q* < 0.1 that 90% of the mutations below the threshold were clustered together, that is, not occurring by chance solely. Subsequently, SigProfilerClusters^49^ with default parameters was subsequently employed to partition the clustered mutations from non-clustered mutations and then to further subclassify all clustered mutations into (1) doublet-base substitutions (DBS); (2) multi-base substitutions (MBS); (3) diffuse hypermutation (omikli), defined as the mutational events that are greater than 1 bp but less than the sample-specific IMD cutoff; and (4) longer events (kataegis)^45,50^.

### Tumor mutation burden estimation

Tumor mutation burden (TMB) was defined as the number of nonsynonymous somatic mutations per megabase (muts/Mb) within the coding region of the captured exome (35.618 Mb for the kit used in our study). Likewise, the clustered TMB was determined as the number of clustered nonsynonymous somatic mutations per megabase within the captured coding region. Nonsynonymous mutations were defined as missense, nonsense, nonstop, splice site, translation start site mutations, in-frame and frameshift insertions and deletions.

### Estimation of homologous recombination deficiency (HRD) score

We calculated the HRD score by summing three independent scores, telomeric allelic imbalance (NtAI), LOH, and large-scale state transition (LST), based on Allele-Specific Copy Number Analysis of Tumors (ASCAT) according to previous studies^51,52^. In brief, the NtAI score was defined as the number of subchromosomal regions (longer than 11 Mb) with allelic imbalance extending to the telomere. The LOH score was the number of LOH regions longer than 15 Mb but shorter than the whole chromosome. LOH regions located on chromosome 17 were not included. The LST score was the number of break points between two chromosomal regions longer than 10 Mb after smoothing regions shorter than 3 Mb.

### SCNA signature analysis

SigProfiler was also applied to decipher the CNA signatures^34^. Specifically, CNVMatrixGenerator was first used to categorize the CNA segments into three heterozygosity states, namely heterozygous (CN = { > 0,>0}), loss of heterozygosity (CN = { > 0,0}) and homozygous deletion (CN = {0,0}), taking into account the ploidy, copy number, and segment size. SigProfilerSingleSample was subsequently employed to decipher the CNA signatures.

### Calculation of neoantigens

We first used POLYSOLVER^53^ to infer the 4-digit HLA genotype from WES data (.bam) of paired normal samples. Then, neoantigens were predicted based on NetMHCpan (v4.0)^54^, with the somatic mutation data and HLA genotype data as the inputs. We predicted neoantigens derived from protein coding single nucleotide variants (missense mutations) and small insertions and deletions (INDEL) (frameshift and in-frame indel) separately. Neoantigens were defined as mutations predicted to produce peptide with affinity < 500 nM and of which the corresponding gene was expressed greater than Combat value 1 (evaluated based on median expression rather than the specific sample). We referred to pVAC-seq and made some modifications based on the features of our dataset to construct this algorithm^55^.

### Estimation of HLA-I status

HLA-I status comprises two aspects, namely germline homogeneity and HLA-I LOH. Based on the exome data of paired normal samples from TNBC patients, POLYSOLVER was used to determine the four-digit HLA genotype of each sample^53^. HLA-I germline homogeneity was considered in patients with the same genotype at any one of the HLA-A, HLA-B, or HLA-C loci; if not, HLA-I germline heterogeneity was defined. We used ASCAT-adjusted copy number values (nMajor and nMinor) at the segment level to estimate HLA-I LOH. Copy number values in ASCAT are adjusted by tumor purity. When one of two alleles at any of the main HLA-I loci (HLA-A, HLA-B, and HLA-C) equaled zero, the patient was considered HLA-I LOH; otherwise, HLA-I non-LOH.

### Estimation of intra-tumoral heterogeneity (ITH)

We used ASCAT to estimate the purity and ploidy of each tumor based on the copy number data with the data on somatic mutations using default parameters. A modified PyClone workflow was then adopted to estimate the cancer cell fractions (CCF) of each sample^56^. The fraction of subclonal cancer cells was set as indicators representing the ITH.

### PAM50 classification

We determined the PAM50 subtype of each patient based on the PAM50 classifier as previously described^57,58^. First, the mRNA-seq data (Supplementary Data 1) were subsampled so that the distribution of IHC subtypes of the samples was consistent with the training set used for PAM50. Second, we adjusted the FPKM data to the median gene expression calculated from the PAM50 gene level of the IHC balanced subset. Finally, PAM50 typing was performed as previously described.

### Gene set enrichment analysis (GSEA)

GSEA analysis was run to identify the enriched pathways and interpret transcriptomic data^59^. Pathways were defined by the gene set file Human_GOBP_AllPathways_no_GO_iea_May_05_2019_symbol.gmt that is regularly updated and maintained by the Bader laboratory. GSEA was performed with the geneset size limited to a range between 10 and 300 and 2000 permutations. Gene sets with false discovery rate (FDR) < 0.25 and nominal *P* value < 0.05 were considered significant. We then visualized the pathway network based on the EnrichmentMap App (v.3.3) in Cytoscape (v.3.9.1). Pathways clusters were defined and annotated using a Cytoscape app AutoAnnotate (v.1.3.5).

### Tumor microenvironment (TME) constitution

The infiltration levels of immune cells were inferred as previously described^6^. First, we established a reference compendium that included 364 genes representing 24 TME cell types. The gene set were curated from CIBERSORT (22 immune cells) and MCP-counter (2 stromal cells, including fibroblasts and endothelial cells). Then, we used single-sample gene set enrichment analysis (ssGSEA, “GSVA” function in R) to estimate the immune infiltration based on the transcriptomic data.

### Calculation of literature-defined immune signatures, I-SPY2 immunotherapy-response signatures, and metabolic signatures

We curated literature-defined immune signatures^6^, I-SPY2 immunotherapy-response signatures^36^, and metabolic signatures^7^ to infer the status of tumor immune microenvironment and metabolic signaling (Supplementary Tables 3, 4). According to a previous study^36^, we then calculated the signature score based on the following steps: (1) mean center by genes across all samples; (2) average over genes; (3) Z-score.

### Estimation of mRNA subtype of TNBC

To determine the optimal number of TNBC subtypes, we ran consensus cluster analysis (“ConsensusClusterPlus” package in R) with the expression profile. TNBC samples were classified into four distinct subtypes using K-means clustering (“kmeans” function in R) based on genes with top 2000 standard deviations. TNBC is generally classified as luminal androgen receptor (LAR), immunomodulatory (IM), basal-like immune-suppressed (BLIS), and mesenchymal (MES). Detailed methods of the expression-based TNBC clustering was described previously^3^.

### Estimation of immune subtype of TNBC

A TNBC immune subtype was estimated based on the constituent pattern of each microenvironment cell subset. We conducted NbClust (“NbClust” function in R, index 1⁄4 “all”) testing to identify the optimal number of stable TNBC immune subtypes. Following that, k-means clustering (kmeans in R) was used to separate each TNBC immune subtype according to the putative optimal number of microenvironment clusters based on Nbclust testing. The detailed estimation of TNBC immune subtype was described in a previous study^6^.

### Estimation of metabologenomic subtype of TNBC

In this study, metabolic pathways have been downloaded from the KEGG^60^. A KEGG classification system was used to group pathways into ten major categories. We calculated GSVA enrichment scores for each metabolic pathway using transcriptomic data in each sample. To determine the optimal number of stable metabolic pathway-based TNBC subtypes, we conducted k-means clustering, consensus clustering, and NbClust testing. Each sample was scaled prior to clustering in order to determine the constituent pattern of each metabolic pathway. K-means clustering was assessed using consensus clustering. In this study, the number of clusters that most testing methods support was determined by NbClust (Euclidean distance, k-means clustering, from 2 to 8 clusters). An earlier study described detailed clustering processes^7^.

### Classification of polar metabolite and lipid

Using the Kyoto Encyclopedia of Genes and Genomes (KEGG) database, we classified polar metabolites by their KEGG metabolic pathways^60^. Eight classifications were determined: lipids, amino acids, carbohydrates, nucleotides, peptides, vitamins and cofactors, xenobiotics and others. Our determination of lipid categories and main classes was based on the LIPID MAPS Structure Database (LMSD). We detected five of eight classical lipid categories (fatty acyls [FA], glycerolipids [GL], glycerophospholipids [GP], sphingolipids [SP], sterol lipids [ST]).

### DA score

DA scores reflect the tendency for pathways to have higher levels of metabolites than control groups^5,61^. A nonparametric DA test (in this study, Mann–Whitney U tests) is performed on all metabolites in a pathway before calculating the score. Once the metabolites have been determined as significantly increased or decreased in abundance, the DA score is calculated as: (number of metabolites increased - number of metabolites decreased)/number of measured metabolites within the pathway. DA scores range from −1 to 1. When the pathways are scored as −1, it indicates that all metabolites decreased in abundance, while when they are scored as 1, all increased in abundance.

### Estimation of metabolite subtype of TNBC

Data from both lipids/metabolites were pre-processed before being clustered by SNF^62^. Our study focused on lipids and metabolites that showed significant tumor-normal differences (FDR < 0.01; |log2 fold change | > 1). We further filtered these lipids/metabolites with standard deviation (SD). To cluster downstream SNFs, we retained metabolites with the top 200 SDs and lipids with the top 400 SDs. An optimal number of clusters of three was determined using the function “estimateNumberOfClustersGivenGraph” in R package “SNFtools” (both Eigen-gap best and rotation cost best). Check the previous study for method details^5^.

### Statistical analysis

Comparative analyses were performed between patients with and without calcifications of high suspicion for malignancy. The Mann-Whitney Wilcoxon test and the Kruskal–Wallis test were applied to analyze the continuous variables, while Pearson’s chi-square test and Fisher’s exact test were utilized to compare the categorical variables. Multivariate logistics regression was also used to adjust confounding bias when performing the comparison analysis. We adopted overall survival (defined as the interval between surgery and death from any cause), distant metastasis-free survival (defined as the interval between surgery and first distant metastasis), and relapse-free survival (defined as the interval between surgery and locoregional/distant recurrence and death from any causes), as the main outcomes in our study. Adjusted hazard ratio (HR) and 95% confidence interval (CI) were estimated using the multivariate Cox proportional hazards model. The *P* values were adjusted to the false discovery rate (FDR) using the Benjamini–Hochberg procedure in multiple comparisons. A *P*-value of < 0.05 suggested statistical significance unless otherwise stated. All analyses were performed using R version 4.1.1 (https://cran.r-project.org/).

### Reporting summary

Further information on research design is available in the Nature Portfolio Reporting Summary linked to this article.

## Supplementary information

Supplementary Figures

Supplementary Tables

Reporting summary

Supplementary Data

## Data Availability

The accession number for all data reported in this paper is NODE: OEP000155. These data can be viewed in The National Omics Data Encyclopedia (NODE) (http://www.biosino.org/node) by pasting the accession (OEP000155) into the text search box or through the URL. Microarray data and sequence data have also been deposited in the NCBI Gene Expression Omnibus (OncoScan array; GEO: GSE118527) and Sequence Read Archive (WES and RNA-seq; SRA: SRP157974). Other data could be obtained in the Supplementary Table 2 and reference^3,5^.

## Code availability

Specific code will be made available upon request to Z.M.S.
