# Supplementary material for: Calcifications in triple-negative breast cancer: Molecular features and treatment strategies
Source: NPJ Breast Cancer. 2023 Apr 15;9:26. doi: 10.1038/s41523-023-00531-4 (PMC10105779; doi:10.1038/s41523-023-00531-4)
Supplement: Supplementary file 1 — Supplementary Figures [file 41523_2023_531_MOESM1_ESM.pdf]

Supplementary Information for

Calcifications in Triple-Negative Breast Cancer: Molecular Features and Treatment Strategies

Cai-Jin Lin, Wen-Xuan Xiao, Tong Fu, Xi Jin, Zhi-Ming Shao, Gen-Hong Di

This PDF file includes:

Supplementary Figure 1. Mutational and copy number alteration signatures across different calcification groups

Supplementary Figure 2. Genomic regions with significantly recurrent somatic copy number alterations (SCNA) across different calcification groups.

Supplementary Figure 3. Immune-related features across different calcification groups.

Other Supplementary Information for this manuscript includes the following:

Supplementary Table 1. Baseline characteristics of the FUSCCTNBC-Mammography cohort.

Supplementary Table 2. Clinical and omics annotation of the study cohort.

Supplementary Table 3. Immune-related signatures.

Supplementary Table 4. Metabolism-related signatures.

(Supplementary Tables 1/2/3/4 are provided in a single Excel file.)

Supplementary Data 1. RNA-seq matrix (provided as a separate compressed file).

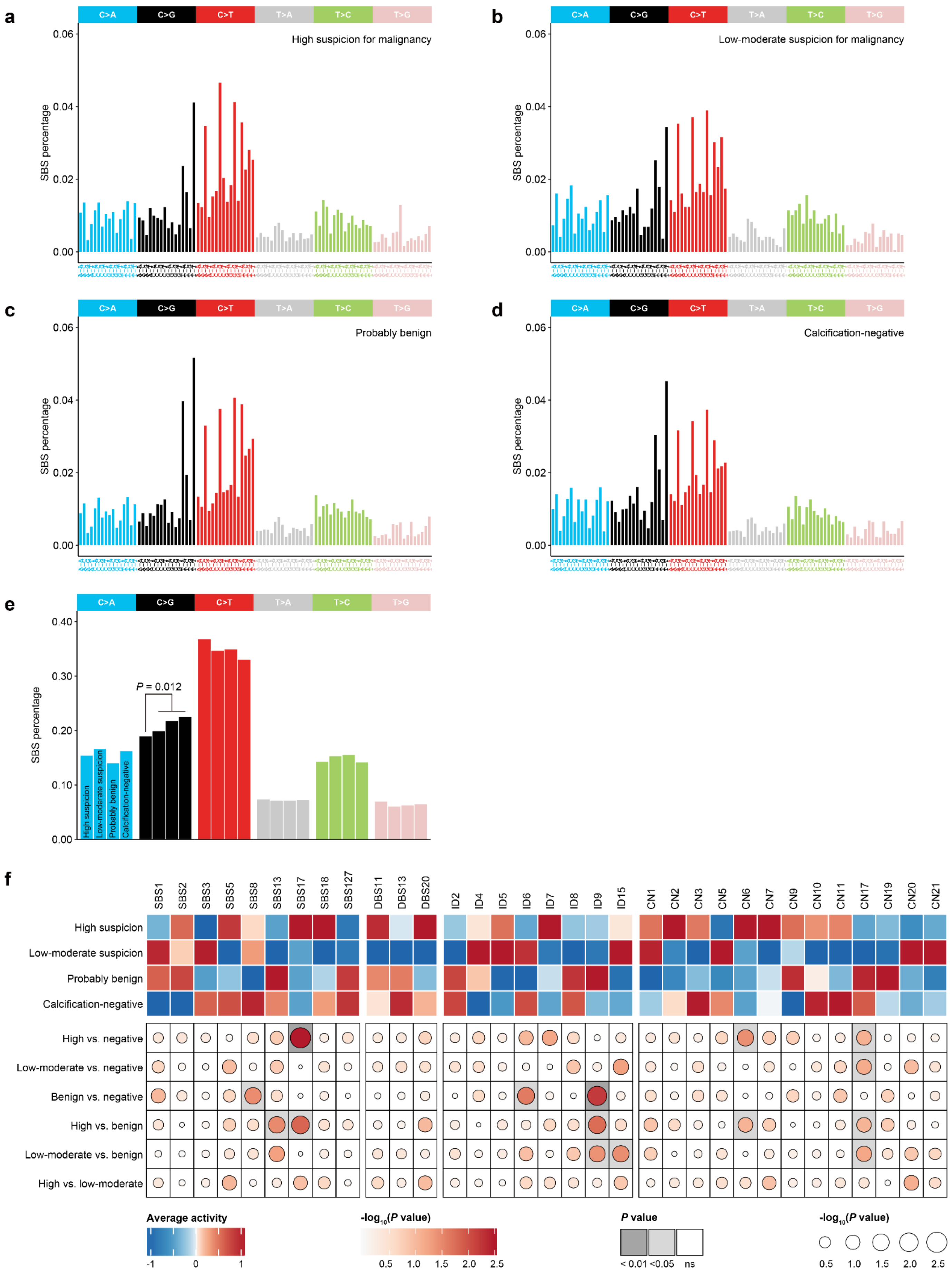

Supplementary Figure 1. Mutational and copy number alteration signatures across different

calcification groups. a-d Mutational spectra of somatic substitutions in tumors with calcifications of high suspicion for malignancy (a) or low-moderate suspicion for malignancy (b), probably benign calcifications (c), and no calcifications (d). The x-axis shows 96 mutation types on a trinucleotide context, colored by base substitution type. **e** Comparison of the somatic substitution contributions across different calcification groups using logistics regression model. **f** Activities of single-base substitution (SBS), doublet-base substitution (DBS), small insertion-deletion (ID), and copy number (CN) signatures across different calcification groups. All *P* values were obtained based on logistics regression models using the gaussian family.

Calcifications of high suspicion for malignancy

Calcifications of low-moderate suspicion for malignancy and probably benign calcifications

Calcification-negative

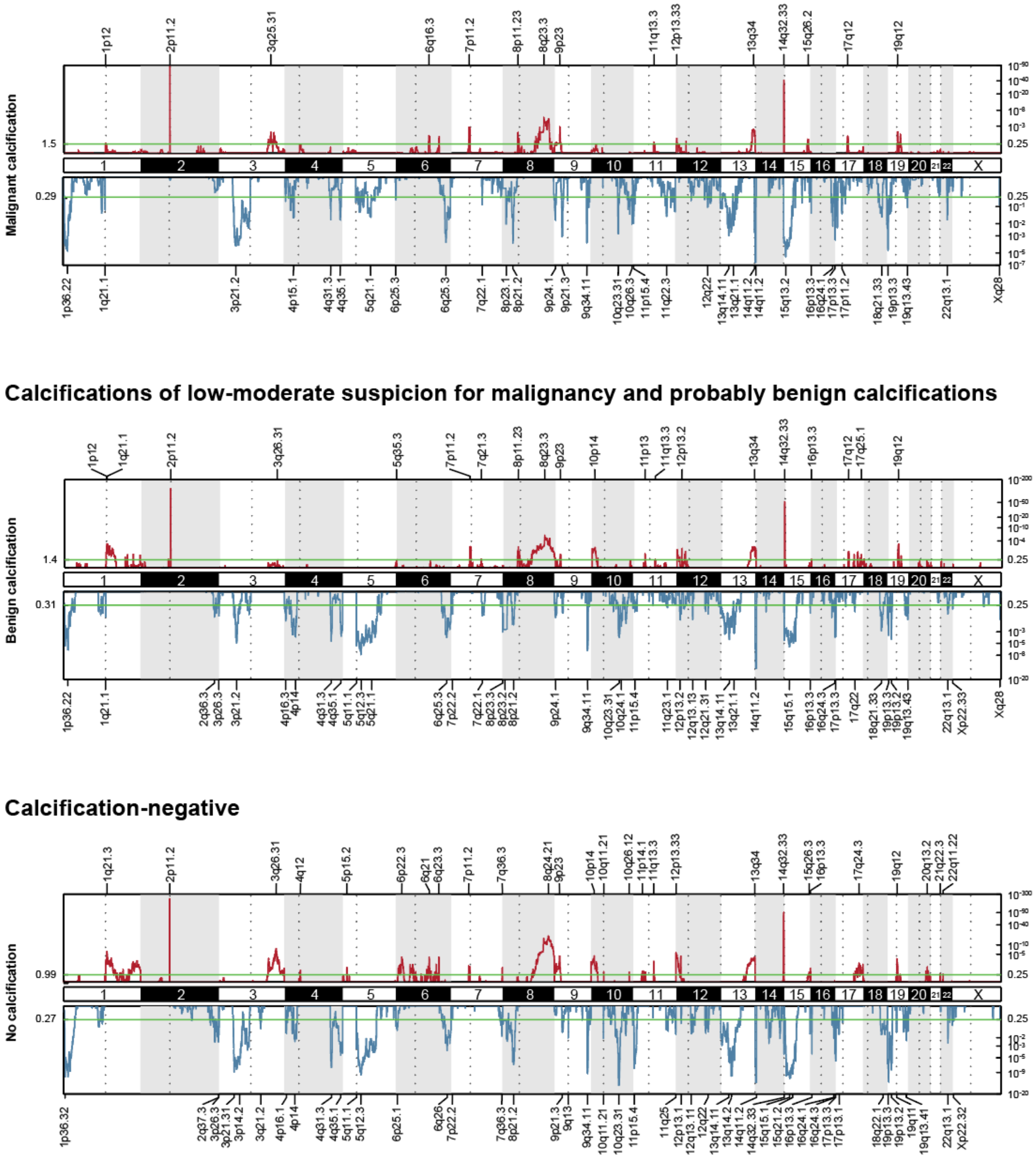

Supplementary Figure 2. Genomic regions with significantly recurrent somatic copy number alterations (SCNA) obtained through GISTIC2 across different calcification groups.

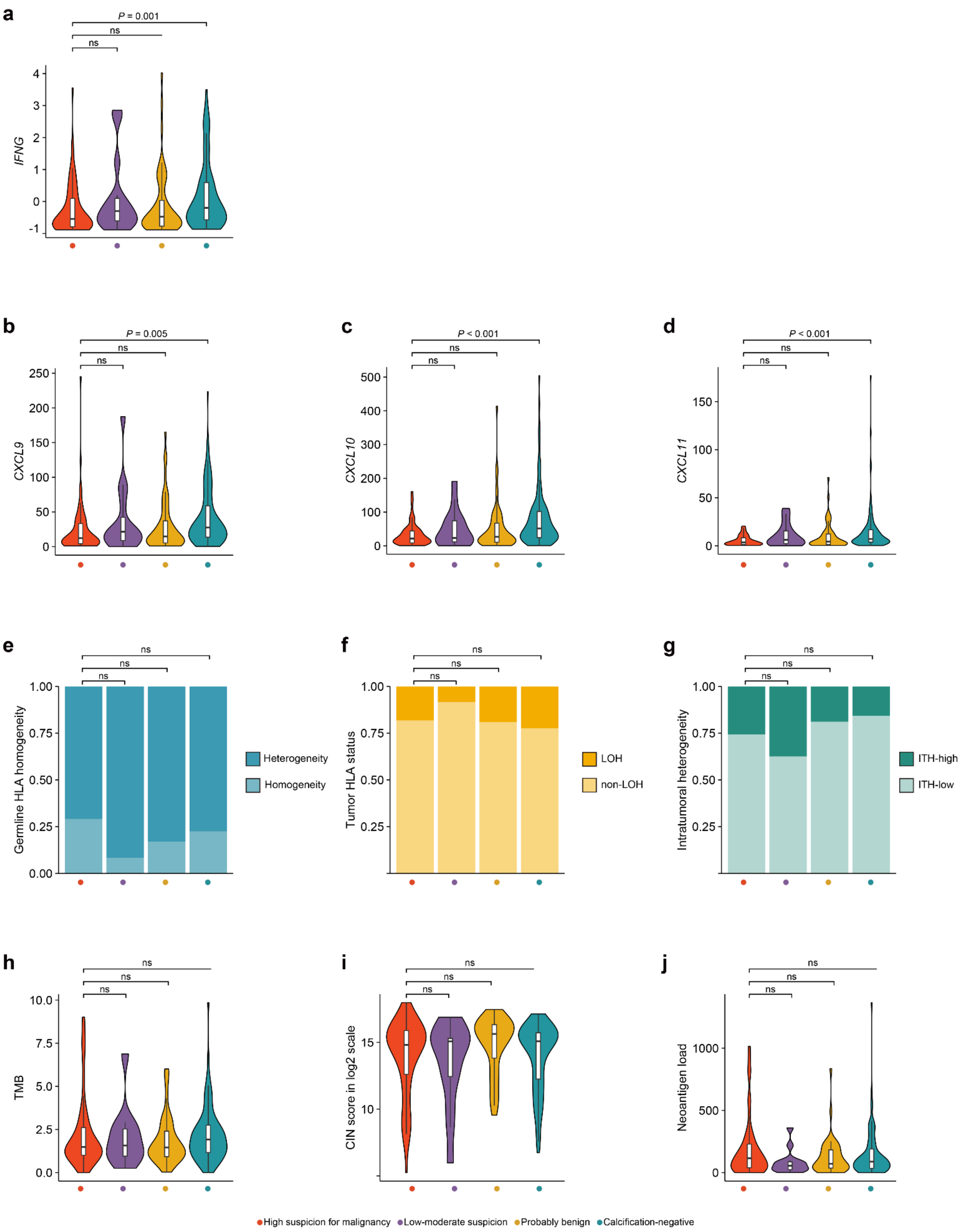

Supplementary Figure 3. Immune-related features across different calcification groups. a Comparison of the expression of *IFNG* across different calcification groups. **b-d** Comparison of the

expression of *CXCL9* (b), *CXCL10* (c), and *CXCL11* (d) across different calcification groups. **e-g** Comparison of germline HLA heterogeneity (e), tumor HLA-LOH status (f), and intra-tumoral heterogeneity (g) across different calcification groups. **h-j** Comparison of tumor mutation burden (h), chromosomal instability score (i), and neoantigen load (j) across different calcification groups. For boxplots in **a-d** and **h-j**, the center lines represent median values; the bounds of the boxplot represent the interquartile ranges; the whiskers show the range of the data; the violin plots show the data distribution. All *P* values were obtained based on logistics regression models with the gaussian family used for continuous data (**a-d, h-j**) and the binomial family used for categorical data (**e-g**).
