# Supplementary material for: Calcifications in triple-negative breast cancer: Molecular features and treatment strategies
Source: NPJ Breast Cancer. 2023 Apr 15;9:26. doi: 10.1038/s41523-023-00531-4 (PMC10105779; doi:10.1038/s41523-023-00531-4)
Supplement: Supplementary file 3 — Reporting summary [file 41523_2023_531_MOESM3_ESM.pdf]

nature portfolio

Corresponding author(s): Gen-Hong Di, Zhi-Ming Shao, Xi Jin

Last updated by author(s): Mar 23, 2023

Reporting Summary

Nature Portfolio wishes to improve the reproducibility of the work that we publish. This form provides structure for consistency and transparency in reporting. For further information on Nature Portfolio policies, see our Editorial Policies and the Editorial Policy Checklist.

Statistics

For all statistical analyses, confirm that the following items are present in the figure legend, table legend, main text, or Methods section.

n/a	Confirmed	
☐	☒	The exact sample size (n) for each experimental group/condition, given as a discrete number and unit of measurement
☐	☒	A statement on whether measurements were taken from distinct samples or whether the same sample was measured repeatedly
☐	☒	The statistical test(s) used AND whether they are one- or two-sided<br>*Only common tests should be described solely by name; describe more complex techniques in the Methods section.*
☐	☒	A description of all covariates tested
☐	☒	A description of any assumptions or corrections, such as tests of normality and adjustment for multiple comparisons
☐	☒	A full description of the statistical parameters including central tendency (e.g. means) or other basic estimates (e.g. regression coefficient) AND variation (e.g. standard deviation) or associated estimates of uncertainty (e.g. confidence intervals)
☐	☒	For null hypothesis testing, the test statistic (e.g. F, t, r) with confidence intervals, effect sizes, degrees of freedom and P value noted<br>*Give P values as exact values whenever suitable.*
☒	☐	For Bayesian analysis, information on the choice of priors and Markov chain Monte Carlo settings
☒	☐	For hierarchical and complex designs, identification of the appropriate level for tests and full reporting of outcomes
☒	☐	Estimates of effect sizes (e.g. Cohen's d, Pearson's r), indicating how they were calculated

Our web collection on statistics for biologists contains articles on many of the points above.

Software and code

Policy information about availability of computer code

Data collection	Data collection were reported in our previous study titled "Genomic and Transcriptomic Landscape of Triple-Negative Breast Cancers". For sample processing, quality control were performed by checking the percentage of tumor cells under microscope. All samples have 50% or more of tumor cells. DNA was isolated with TGuide M24 (Tiangen, Beijing, China) and performed quality control by measuring absorbance at 260 nm (A260) and 280 nm (A280) with a NanoDrop 2000 spectrophotometer (Thermo Scientific, Wilmington, DE, USA). RNA was purified with MiRNeasy mini kit (Qiagen, Hilden, Germany), stored in RNA-later solution and performed quality control similar as DNA. For WES data, DNA from tumor tissues and paired white blood cells of 279 samples were prepared for WES. DNA was fragmented on a Bioruptor Plus sonication system and sequenced on an Illumina HiSeq X TEN platform (Illumina Inc., San Diego, CA, USA). BWA-mem, Sentieon tools, VarScan2, TNseq and TNscope were utilized to generated the mutational profile. A panel of normal (PON) samples filtering, processSomatic and somaticFilter tools were used to improve the specificity in mutation calling (coverage≥10, VAF≥0.2). In addition, mutations in at least two out of three callers (TNseq, TNscope and VarScan2) were included to construct the final somatic mutation compendium. For SCNA data, DNA from tumor tissues of 335 samples were conducted with SCNVs sequencing. Genome-wide somatic copy number analysis was performed based on the protocol of the OncoScan CNV Assay Kit (Affymetrix, Santa Clara, CA, USA). Cluster intensity values were automatically calculated using a built-in algorithm from DAT files using GeneChip Command Console software (Affymetrix, Inc.) to generate a CEL file. OncoScan Console 1.3 software (Affymetrix, Inc.) and GISTIC2.0 (v2.0.22) were used to generate peak level and gene level copy number values. ASCAT algorithm was used to adjust copy numbers of genes based on ploidy and purity. For RNA data, RNA sequencing was performed on 245 samples. RNA library was prepared as described in the Illumina TruSeq Stranded Total RNA LT sample preparation kit. Then the libraries were sequenced on the Illumina HiSeq X TEN platform (Illumina Inc., San Diego, CA, USA). Tophat-cufflinks pipeline (reference: Hg19, GRCh37_snp_tran) were utilized to generate per million mapped fragments (FPKM) data. HTA 2.0 array sequencing was performed on the other 141 samples. Detailed information of HTA data was described in our previous article. We utilized Combat ("ComBat" function in R) to adjust batch effects between the RNA-seq and HTA array datasets. To construct the expression profile with relatively accurate values, we removed non-protein coding genes and genes whose FPKMs were not 0 in more than 30% samples before Combat

Data analysis

Mutational matrices of SBS96, DBS78, and ID83 based on the somatic mutations and their immediate sequence context were first created using SigProfilerMatrixGenerator with default parameters. We employed SigProfiler, including SigProfilerMatrixGenerator, SigProfilerExtractor, SigProfilerSimulator, SigProfilerClusters, CNVMatrixGenerator, and SigProfilerSingleSample, to extract mutational signatures, CNA signatures, and clustered mutations across tumor samples in the FUSCCTNBC-Mammography cohort with default parameters. We calculated the HRD score by summing three independent scores, telomeric allelic imbalance (NtAI), LOH, and large-scale state transition (LST), based on Allele-Specific Copy Number Analysis of Tumors (ASCAT). We first used POLYSOLVER59 to infer the 4-digit HLA genotype from WES data (.bam) of paired normal samples. Then, neoantigens were predicted based on NetMHCpan (v4.0), with the somatic mutation data and HLA genotype data as the inputs. We predicted neoantigens derived from protein coding single nucleotide variants (missense mutations) and small insertions and deletions (INDEL) (frameshift and in-frame indel) separately. Neoantigens were defined as mutations predicted to produce peptide with affinity < 500 nM and of which the corresponding gene was expressed greater than Combat value 1 (evaluated based on median expression rather than the specific sample). We referred to pVAC-seq and made some modifications based on the features of our dataset to construct this algorithm. We used ASCAT to estimate the purity and ploidy of each tumor based on the copy number data with the data on somatic mutations using default parameters. A modified PyClone workflow was then adopted to estimate the cancer cell fractions (CCF) of each sample. The fraction of subclonal cancer cells was set as indicators representing the ITH. ssGSEA was used to infer tumor microenvironment (TME) constitution. To determine the optimal number of TNBC subtypes, we ran consensus cluster analysis (“ConsensusClusterPlus” package in R) with the expression profile. TNBC samples were classified into four distinct subtypes using K-means clustering ("kmeans" function in R) based on genes with top 2000 standard deviations. A TNBC immune subtype was estimated based on the constituent pattern of each microenvironment cell subset. We conducted NbClust (“NbClust” function in R, index 1⁄4 “all”) testing to identify the optimal number of stable TNBC immune subtypes. Following that, k-means clustering (kmeans in R) was used to separate each TNBC immune subtype according to the putative optimal number of microenvironment clusters based on Nbclust testing. The detailed estimation of TNBC immune subtype was described in a previous study.In this study, metabolic pathways have been downloaded from the KEGG. A KEGG classification system was used to group pathways into ten major categories. We calculated GSVA enrichment scores for each metabolic pathway using transcriptomic data in each sample. To determine the optimal number of stable metabolic pathway-based TNBC subtypes, we conducted k-means clustering, consensus clustering, and NbClust testing. Each sample was scaled prior to clustering in order to determine the constituent pattern of each metabolic pathway. K-means clustering was assessed using consensus clustering. In this study, the number of clusters that most testing methods support was determined by NbClust (Euclidean distance, k-means clustering, from 2 to 8 clusters). An earlier study described detailed clustering processes. To infer the metabolite subtype of TNBC, data from both lipids/metabolites were pre-processed before being clustered by SNF. Our study focused on lipids and metabolites that showed significant tumor-normal differences (FDR < 0.01; |log2 fold change| > 1). We further filtered these lipids/metabolites with standard deviation (SD). To cluster downstream SNFs, we retained metabolites with the top 200 SDs and lipids with the top 400 SDs. An optimal number of clusters of three was determined using the function “estimateNumberOfClustersGivenGraph” in R package “SNFtools” (both Eigen-gap best and rotation cost best). Check the previous study for method details. All analyses were performed using R version 4.1.1 (https://cran.r-project.org/).

For manuscripts utilizing custom algorithms or software that are central to the research but not yet described in published literature, software must be made available to editors and reviewers. We strongly encourage code deposition in a community repository (e.g. GitHub). See the Nature Portfolio guidelines for submitting code & software for further information.

Data

Policy information about availability of data

All manuscripts must include a data availability statement. This statement should provide the following information, where applicable:

- Accession codes, unique identifiers, or web links for publicly available datasets
- A description of any restrictions on data availability
- For clinical datasets or third party data, please ensure that the statement adheres to our policy

The accession number for all data reported in this paper is NODE: OEP000155. These data can be viewed in The National Omics Data Encyclopedia (NODE) (http://www.biosino.org/node) by pasting the accession (OEP000155) into the text search box or through the URL: http://www.biosino.org/node/project/detail/OEP000155. Microarray data and sequence data have also been deposited in the NCBI Gene Expression Omnibus (OncoScan array; GEO: GSE118527) and Sequence Read Archive (WES and RNA-seq; SRA: SRP157974). Other data could be obtained in the Supplementary Table 2

Human research participants

Policy information about studies involving human research participants and Sex and Gender in Research.

Reporting on sex and gender

All patients included in our study were female, as reported by the patients themselves. We have indicated this in the revised submission and added the gender information in Supplementary Table 4. Accordingly, we did not perform sex- and gender-based analyses.

Population characteristics

We performed multi-omics sequencing for a total of 312 patients. The clinical data, including demographics, postoperative pathology, treatment regimen, and follow-up, were recorded in detail. All women underwent surgery between 2007 and 2014. We then updated the follow-up data on June 30, 2021. Clinical records (N = 312) and mammographic images (N = 312) were collected in detail, coupled with hematoxylin and eosin (H&E) and immunohistochemical (IHC) staining slides (N = 159), whole exome sequencing (WES; N = 198), OncoScan (N = 265), transcriptomics (N = 249), lipidomics and polar metabolomics (N = 216) data. Clinicopathological characteristics were presented in Supplementary Table 1.

Recruitment

All women underwent surgery between 2007 and 2014. We then updated the follow-up data on June 30, 2021. The inclusion criteria were listed as follows: (1) female patients diagnosed with unilateral invasive breast carcinoma; (2) pathological examination of tumor samples examined at the Department of Pathology of FUSCC (ER, PR, and HER2 statuses individually evaluated by two experienced pathologists based on IHC analysis and fluorescence in situ hybridization). Tumor specimens were classified into breast cancer subtypes based on ER and PR status and the HER2 IHC and/or FISH results rendered at the time of diagnosis according to the American Society of Clinical Oncology (ASCO) and College of American Pathology (CAP)

	guideline recommendations; (3) sufficient fresh tissue available for further research.
Ethics oversight	All tissue samples included in this study were obtained after the approval of our research by the FUSCC Ethics Committee, and each patient provided written informed consent prior to participation.

Note that full information on the approval of the study protocol must also be provided in the manuscript.

Field-specific reporting

Please select the one below that is the best fit for your research. If you are not sure, read the appropriate sections before making your selection.

☒ Life sciences ☐ Behavioural & social sciences ☐ Ecological, evolutionary & environmental sciences

For a reference copy of the document with all sections, see nature.com/documents/nr-reporting-summary-flat.pdf

Life sciences study design

All studies must disclose on these points even when the disclosure is negative.

Sample size	We performed multi-omics sequencing for a total of 312 patients undergoing surgery between 2007 and 2014.
Data exclusions	The exclusion criteria were pre-established that samples without sufficient tissue for sequencing by core needle biopsy or data which failed sequencing quality control were excluded before enrollment. Besides, cases of carcinomas in situ or inflammatory breast cancer and patients with de novo stage IV breast cancer were excluded.
Replication	No experimental validation was performed in our study.
Randomization	No randomization was performed in our study for all participants were enrolled and studied in the common procedure.
Blinding	No bliding was performed in our study for all participants were enrolled and studied in the common procedure.

Reporting for specific materials, systems and methods

We require information from authors about some types of materials, experimental systems and methods used in many studies. Here, indicate whether each material, system or method listed is relevant to your study. If you are not sure if a list item applies to your research, read the appropriate section before selecting a response.

Materials & experimental systems

n/a	Involved in the study	
☒	☐	Antibodies
☒	☐	Eukaryotic cell lines
☒	☐	Palaeontology and archaeology
☒	☐	Animals and other organisms
☐	☒	Clinical data
☒	☐	Dual use research of concern

Methods

n/a	Involved in the study	
☒	☐	ChIP-seq
☒	☐	Flow cytometry
☒	☐	MRI-based neuroimaging

Clinical data

Policy information about clinical studies

All manuscripts should comply with the ICMJE guidelines for publication of clinical research and a completed CONSORT checklist must be included with all submissions.

Clinical trial registration	It is not a clinical trial study.
Study protocol	It is not a clinical trial study.
Data collection	It is not a clinical trial study.
Outcomes	We adopted overall survival (OS; defined as the interval between surgery and death from any cause), distant metastasis-free survival (DMFS; defined as the interval between surgery and first distant metastasis), and relapse-free survival (RFS; defined as the interval between surgery and locoregional/distant recurrence and death from any causes), as the main outcomes in our study.
